# Micro-scale aerosol jet printing of superparamagnetic Fe_3_O_4_ nanoparticle patterns

**DOI:** 10.1038/s41598-022-22312-y

**Published:** 2022-10-26

**Authors:** Silvia Taccola, Tomas da Veiga, James H. Chandler, Oscar Cespedes, Pietro Valdastri, Russell A. Harris

**Affiliations:** 1 Future Manufacturing Processes Research Group, University of Leeds, Leeds, UK; 2 STORM Lab, University of Leeds, Leeds, UK; 3 School of Physics and Astronomy, University of Leeds, Leeds, UK

**Keywords:** Materials for devices, Surface patterning, Magnetic properties and materials

## Abstract

The opportunity to create different patterns of magnetic nanoparticles on surfaces is highly desirable across many technological and biomedical applications. In this paper, this ability is demonstrated for the first time using a computer-controlled aerosol jet printing (AJP) technology. AJP is an emerging digitally driven, non-contact and mask-less printing process which has distinguishing advantages over other patterning technologies as it offers high-resolution and versatile direct-write deposition of a wide range of materials onto a variety of substrates. This research demonstrates the ability of AJP to reliably print large-area, fine-feature patterns of superparamagnetic iron oxide nanoparticles (SPIONs) onto both rigid material (glass) and soft and flexible materials (polydimethylsiloxane (PDMS) films and poly-L-lactic acid (PLLA) nanofilms). Investigation identified and controlled influential process variables which permitted feature sizes in the region of 20 μm to be realised. This method could be employed for a wide range of applications that require a flexible and responsive process that permits high yield and rapid patterning of magnetic material over large areas. As a first proof of concept, we present patterned magnetic nanofilms with enhanced manipulability under external magnetic field gradient control and which are capable of performing complex movements such as rotation and bending, with applicability to soft robotics and biomedical engineering applications.

## Introduction

Magnetic iron oxide nanoparticles have been gaining major attention because of their wide variety of potential applications in diverse fields such as biomedicine, catalysis, energy, and environmental monitoring^1–7^. Within this framework, spatial arrangement of magnetic nanoparticles in well-defined patterns across a substrate is often needed to achieve specific desired functions. This is highlighted in several applications, but the development of an efficient and effective manufacturing method for controlled patterning of magnetic nanoparticles on surfaces remains a significant challenge^8–13^. The combinations of lithographic techniques and convective self-assembly can be used to address some of the issues; different template-driven manufacturing processes, including photo and electro-beam lithography^8^, soft lithography^9,10^, and dip-pen nanolithography^11^ have been used to date to generate patterns of magnetic structures with dimensions in the sub-100 nm to micrometre length scale. However, there are some inherent limitations associated with these methods, including the need for multiple processing steps and complex instrumentation making them slow and costly, and their template-based nature making mass customisation and iterative, high-yield and flexible production unfeasible. Alternatively, direct-write techniques, such as ink-jet printing^12^ and laser direct writing^13^ are attractive due to their characteristics of greater simplicity, design flexibility, fast-prototyping and material saving. However, in their conventional format, they offer limited printing resolution with a minimum feature size in the range of 50–100 µm^12^.

This research proposes the use of Aerosol Jet Printing (AJP) as an enabling manufacturing process which could introduce new possibilities of producing magnetic patterns at micron-scales onto different substrates. AJP is an emerging contactless direct write technology that have been explored in a wide range of applications for the digital manufacturing of electronic components, actuators, sensors and structured surfaces for tissue engineering^13–17^. The working principle of AJP is the use of a focussed aerosol for the high-resolution printing (down to 10 μm) of a variety of materials at nozzle–substrate offsets of 1–5 mm, allowing for patterning over existing structures, different surface textures, across curved surfaces, and into channels^18–20^. Depending on the viscosity of the ink and the required printing performance for the application, ultrasonic or pneumatic atomization can be used, allowing the printing of liquid materials with a wide viscosity range (1–1000 cP). Examples of materials used to date include polymers, metal nanoparticles, ceramics, and proteins^21–27^. In the field of magnetic materials, Craton et al. recently reported the use of AJP for the deposition of nickel-zinc ferrite nanoparticles/polyimide nanocomposites for microwave packaging applications^28^.

In this presented work superparamagnetic iron oxide nanoparticles (often referred to as SPIONs) were selected to investigate AJP for micro-scale deposition of magnetic patterns onto different substrates. Among the magnetic materials, SPIONs are of significant interest in biological and biomedical applications due to their high biocompatibility and low toxicity^29,30^. These properties, together with their high magnetic susceptibility, their high saturation magnetization, and their ability to convert electromagnetic energy into heat under an alternating magnetic field, are highly relevant in applications such as drug delivery^31^, hyperthermia^32^, biosensing^33^, bioimaging^30^, tissue engineering^34^ and remotely controlled micro-/nanodevices for minimally invasive medicine^35,36^. Microdevices with surface-decorated SPIONs demonstrated to date include microgrippers, microswimmers, and microrobots for imaging-guided therapy^37–39^.

**Figure 1:**
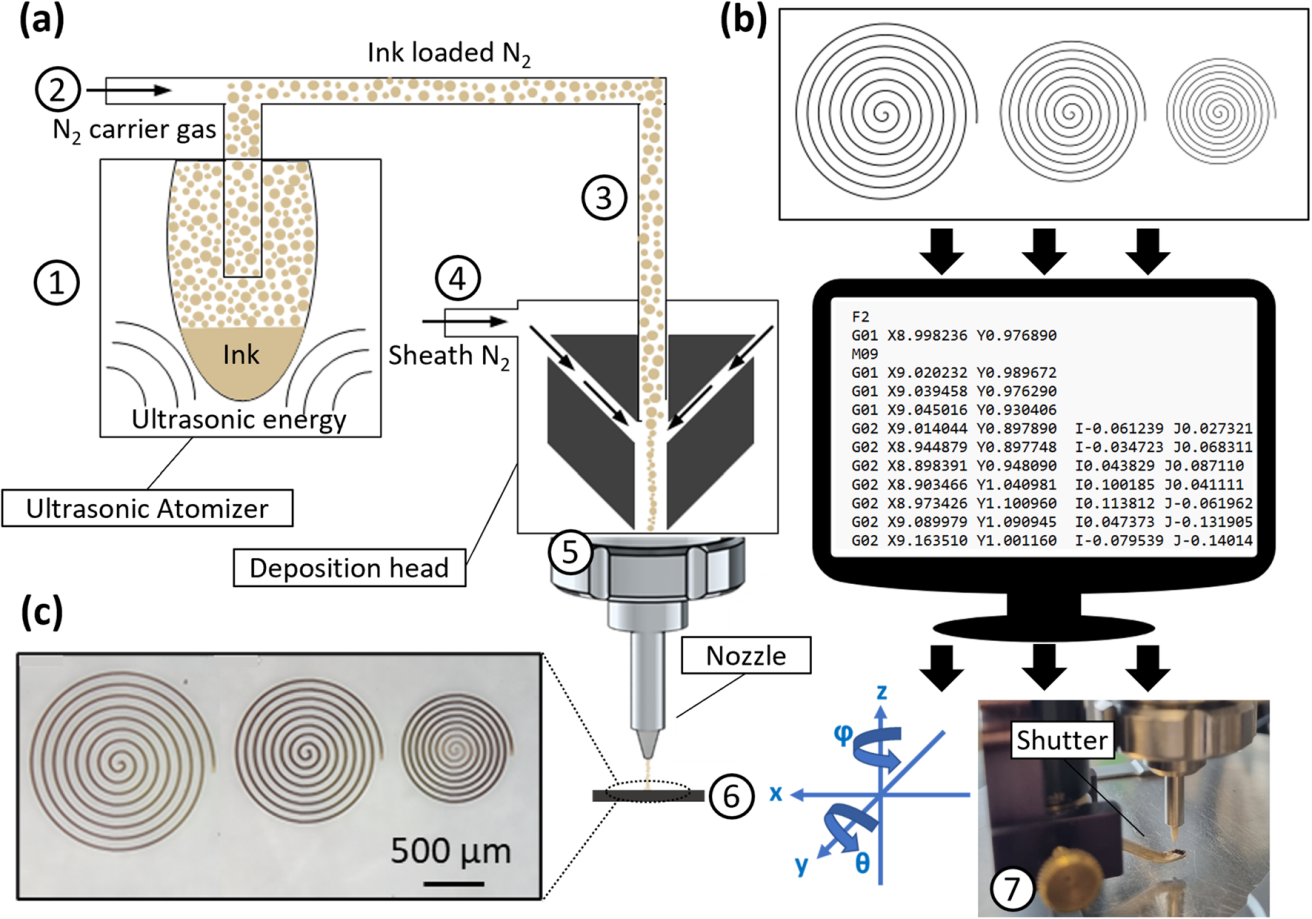
(**a**) A schematic of the AJP process using an ultrasonic atomiser. (1) The material, formulated as an ink, is ultrasonically atomised. (2) An inert gas (N_2_) is used to increase the pressure in the atomiser chamber. (3) The aerosol is transported to the deposition head with the carrier gas. (4) The aerosol is focused and accelerated by a further annular sheath of inert gas. (5) The resulting high velocity jet is deposited onto the substrate through the nozzle. (6) The automated stage is moved to produce a pattern. (7) On/off patterning is achieved by interrupting the jet with a mechanical shutter. (**b**) Program containing manipulation instructions is generated from digital design data (**c**) Magnetic material with the desired pattern is deposited directly onto the substrate surface.

A schematic of the AJP apparatus and process used in this work to freely deposit micro-scale SPION patterns on different substrates is illustrated in Fig. 1. Commercially available SPIONs were dispersed in suitable liquid carriers, aerosolized using ultrasonic atomization, transported and deposited as a focused stream onto different substrates, and then fixed using a drying step (Fig. 1a). Our AJP apparatus comprises of a bespoke high resolution 5-axis stage which moves the substrate below the aerosol stream under Computer Numerical Control (CNC). The design is created in standard graphics or Computer Aided Design (CAD) software before being translated to machine control code (G-Code) (Fig. 1b). Combining this with the high resolution of the AJP, facilitates patterning ranges of macro to micro scale (Fig. 1c). We demonstrate the use of AJP to reliably produce magnetic micro-scale printed structures in the region of 20 μm wide onto both rigid (glass slides) as well as soft and flexible substrates such as polydimethylsiloxane (PDMS) films and poly-L-lactic acid (PLLA) nanofilms. PDMS was selected as printing substrates because it is of particular interest to soft microfluidics and soft robots^40,41^. PLLA films with sub-micrometric thickness (also called nanofilms or nanosheets) were selected because they have been found to be adaptable to many biomedical applications, such as injectable nano-patches on internal organs surfaces, innovative alternative to traditional wire for suturing wounds in open and minimally invasive surgery, or flexible cell growth supports^42–45^. Within this framework, the use of the AJP to freely deposit micro-scale SPIONs patterns on existing structures of these materials can provide additional functionality and pave the way for new capabilities and applications, ranging from magnetic micro-devices with enhanced locomotion performances to magnetic scaffolds for tissue engineering. As a first proof of concept, PLLA patterned nanofilms with a varied range of micro/milli-scale designs were fabricated. As a consequence of creating asymmetrical patterns of SPIONs, magnetic nanofilms were created which demonstrate enhanced controllability under external magnetic field gradient, showing their ability to perform planned sequential movements consisting of rotations and translations and 2D-to-3D shape morphing by out-of-plane bending.

## Materials and methods

### Fe_3_O_4_ magnetic nanoparticles material preparation

EMG1300M superparamagnetic nanoparticles with polymer-coated surface modification were purchased from FerroTec Co. The particles are a 50/50 mixture of Fe_3_O_4_/γ-Fe_2_O_3_ with an average particle size of 10 nm and a weight percent of iron oxide of 60.0–80.0%. Colloid dispersions of particles are formed by dissolving dry particles in compatible solvents, such as toluene. For the initial determination of a suitable print formulation, a chemical composition experiment was conducted with toluene as the main solvent and terpineol as co-solvent. Three different toluene:terpineol formulations have been tested: 100: 0% v/v, 95: 5% v/v, and 90: 10% v/v. SPIONs concentration was fixed at 20 mg/ml. To achieve a stable colloid, sonicating and heating of the ferrofluid at 35 °C in an ultrasound bath for 30 min was necessary. The viscosity of the resultant material formulations (which we now refer to as inks) was measured using a microfluidic viscometer (MicroVisc, RheoSense, Inc.).

### Microdeposition process

An Optomec Aerosol Jet print engine (Optomec Inc.) was engineered into a programmable 5-axis Cartesian stage controlled through a control code (G-Code) input to Aerotech A3200 Automation Controller, which moves the substrate below the aerosol. The linear translation stages (Thorlabs DDS300/M) provide a minimum incremental movement of 10 nm and a 300 mm travel distance in the XY plane. The prepared magnetic nanoparticles ink was processed in the ultrasonic atomizer of the aerosol-jet printer. Nitrogen was used as the inert sheath and atomiser gas. A 100 µm nozzle, a scanning speed of 2 mm/s and a working distance of 2.5 mm were used throughout. Other machine processing parameters that were varied as part of the investigation included carrier gas flow rate (10, 15, 20 SCCM) and sheath gas flow rate (10, 15, 20, 30, 45, 60, 80 SCCM). Gas flow rates are quoted in standard cubic centimetres per minute (SCCM). Immediately after printing, printed patterns were heated in the oven at 80 °C for 10 min to remove solvent. The test pattern for printing consisted of straight lines 10 mm long. A single deposition pass was used for all prints. Glass slides, polydimethylsiloxane (PDMS) films, and poly-L-lactic acid (PLLA) nanofilms were used as printing substrates.

### Morphological characterization of the printed patterns

For efficient observation and characterization of the printed lines, glass slides were chosen as the substrates for morphological characterization. The ink showed good wetting on glass slides and hence no surface pre-treatment was required. For a preliminary investigation of the printed lines, optical images were taken by Olympus-BX53 microscope (Olympus), covering a magnification range from 2.5× to 50× . The thickness, the width at base, the width at half height, and surface roughness of the magnetic lines were evaluated with a Bruker Dimension Icon atomic force microscope operating in PeakForce tapping mode using a RTESPA-300 probe (Bruker) with an elastic modulus of 20–80 Nm^−1^, a resonance frequency of 200–400 kHz, and an average tip radius of 8 nm. Cross-sectional analysis was performed by scanning the printed line across the edges (maximum scan range 90 µm). Scan data were levelled with the facet level tool to remove sample tilt, and then the line average thickness was evaluated as the difference between the average heights of a region of interest (ROI) selected on the line surface and the average height of the ROI on the glass slide. The thickness error was calculated as the standard deviation of the line height in the AFM scans (Root Mean Square roughness, RMS). As regards width measurements, the mean and standard deviation of the width at base and the width at half height were calculated by analysing three cross-sectional height profiles for each scan data. For roughness measurements, the surface was scanned over 10 μm × 10 μm areas and measures obtained by software analysis.

### PDMS substrate preparation

Films of PDMS (10:1 ratio of base elastomer to curing agent, Sylgard 184 silicone elastomer base and curing agent, Dow Corning Corp.) were cast into a glass petri dish to a thickness of approximately 2 mm, cured at T = 95 °C for 60 min in an oven, and cut in the desired dimension. A subsequent air plasma treatment (PE-25 Plasma System) was applied for 60 s immediately prior the AJP deposition.

### PLLA nanofilms preparation

Free-standing PLLA nanofilms were fabricated by a single step of spin-coated assisted deposition using a sacrificial layer approach^42^: (1) an 1 wt % aqueous solution of poly(vinyl alcohol) (PVA, average Mw = 15,000, MP Biomedicals Europe) was deposited by spin coating (SPIN 150i, Polos) on a glass slide at 3000 rpm for 20 s, forming the sacrificial layer of water-soluble polymer; (2) the deposition of the nanofilm was obtained by spinning a 10 mg mL^−1^ solution of PLLA (Mw = 80,000–100,000, Polysciences Inc.) in chloroform (CHCl_3_) using the same spinning parameters. After each step, the sample was held at 80 °C on a hot plate for 1 min to remove the excess solvent. The prepared nanofilms, with a thickness of approximately 100 nm^42^, were used as the substrate for AJP deposition of magnetic patterns. The ink showed good wetting on PLLA and hence no surface pre-treatment was required. Finally, the glass slide was immersed in water: the PVA sacrificial layer was dissolved, thus releasing a freely suspended patterned nanofilm. Homogeneous magnetic nanofilms (used as a control for magnetic manipulation experiments) were prepared following the same process, adding 20 mg mL^−1^ of SPIONs to the PLLA solution.

### Magnetic characterization

The magnetic behaviour of the aerosol jet printed magnetic patterns was investigated using a superconducting quantum interference measurement device—vibrating sample magnetometer (SQUID-VSM from Quantum Design). The magnetization curves were recorded for pristine SPIONs and for SPIONs printed on top of a polytetrafluoroethylene (PTFE) tape and a 3 mm × 3 mm piece of silicon wafer. The hysteresis loops were measured at 300 K by cyclically applying a magnetic field up to ± 20 kOe.

### Magnetic manipulation of patterned nanofilms

For the manipulation of the PLLA magnetic nanofilms, the dual External Permanent Magnet (dEPM) platform was used^46,47^. This platform consists of two large Permanent Magnets, each mounted at the end effector of a robotic arm, and is able to generate magnetic fields of up to 200 mT and magnetic field gradients up to 500 mT/m. The nanofilms were suspended in water and placed in between the two robotic arms. The nanofilms were manipulated with magnetic gradients of 300 mT/m. For the first set of experiments the films were fixed in place through their centre in order to evaluate their rotation movements. For the second set of experiments, the nanofilms were allowed to freely move on the water and experience both translation and rotation.

## Results and discussion

An important stage in the aerosol jet deposition process is the formulation of a suitable ink with physical properties, such as viscosity and surface tension, which subsequently allow the creation of a dense mist containing small-diameter, homogenous droplets with high adhesion to the deposition substrate. The creation of a suitable aerosol has been shown to be important to achieving printed narrow lines with good edge definition^14^. Within this framework, ultrasonic atomisation was used in the present work because, compared to pneumatic atomization, it creates a denser aerosol mist containing smaller droplets and it is particularly suited for high-resolution applications^48^.

**Figure 2:**
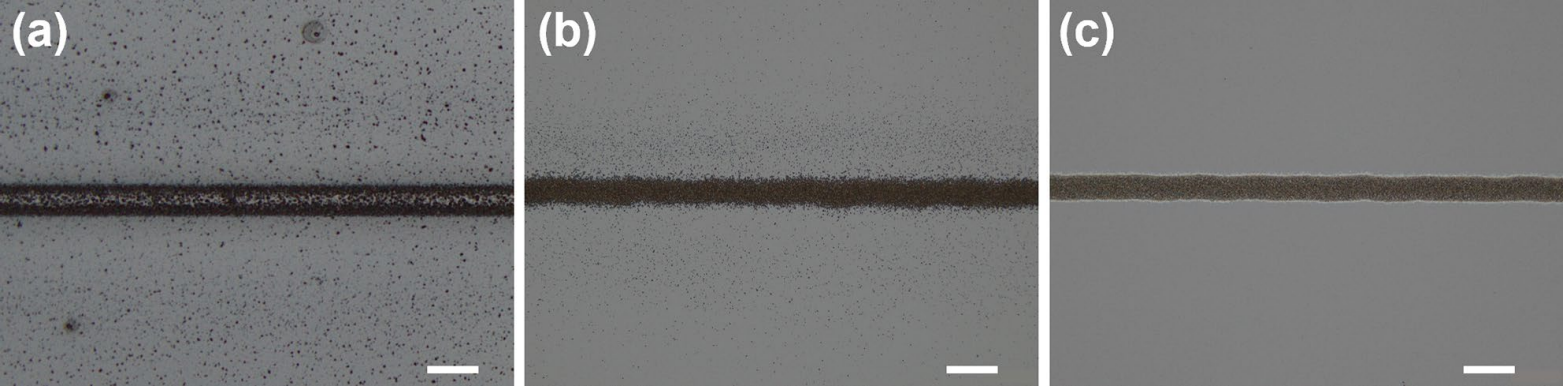
Effect of terpineol on the printed lines: (**a**) No terpineol; (**b**) 5% v/v terpineol; (**c**) 10% v/v terpineol. Printing parameters: 100 µm nozzle, sheath gas flow rate 20 SCCM, carrier gas flow rate 10 SCCM, scanning speed = 2 mm/sec, working distance = 2.5 mm. Scale bar 50 µm.

Ultrasonic atomization allows the deposition of dispersions of functional nanoparticles with a maximum size of 50 nm and a viscosity range of 0–10 cP. In order to prepare an ink compatible with the aerosol printing in terms of viscosity and particle size, EMG1300M were chosen as the functional nanoparticles and toluene as the solvent. Nanoparticles content was chosen to achieve a trade-off between a high concentration of nanoparticles in the ink and the formation of a stable dispersion with low viscosity. In particular, colloidal dispersion of EMG1300M in toluene did not show sedimentation after 24 h from the preparation for a concentration up to 20 mg/ml, reaching a viscosity of 1.86 cP. Although this ink was printable with the AJP, the printed lines suffered from high overspray, with spread of the aerosol deposited beyond the edges (Fig. 2a). This result is in agreement with previous studies which have shown that high-volatility solvents such as toluene evaporate in flight during atomisation, transportation and deposition of the aerosol droplets and, when used alone, result in the deposition of dry particles, producing features with high overspray^14,20^. These also demonstrated that the drying of the particles before deposition can be avoided by including about 10% v/v of a low-volatility co-solvent within the ink^14,20^. For this reason, in the present work terpineol was chosen as the second solvent due to its high viscosity and boiling temperature which make terpineol-based inks among the most efficient in ink-based printing technologies^49^. Two different terpineol concentration were tested (5% and 10% v/v), and the effects of terpineol addition in reducing the spreading of the printed ink on the substrate (i.e. glass slide) are reported respectively in Fig. 2b,c. The solution consisting of 90% v/v toluene and 10% v/v terpineol was then chosen as the solvent for a SPIONs concentration of 20 mg/ml. This resulted in an ink with a viscosity of 3.12 cP which was compatible with AJP system and allowed for print lines with reduced spread and well-defined edges, as show in Fig. 2c.

Key processing variables which control the geometry of the lines printed using the ultrasonic atomizer include the atomization frequency, the carrier gas flow rate which transport the aerosol to the printing head, the sheath gas flow rate which focus the aerosol before deposition, nozzle diameter, stage speed, and the working distance between the substrate and the nozzle. In the case of printing silver nanoparticles, Mahajan et al. previously demonstrated that the key factor affecting line size is the ratio of the sheath and carrier gas flow rates, defined as the focus ratio (FR, Eq. 1)^50^.

1 $$Focus \; Ratio \left( {FR} \right) = \frac{Sheath\; gas\; flow\; rate}{{Carrier\; gas\; flow\; rate}}$$

**Figure 3:**
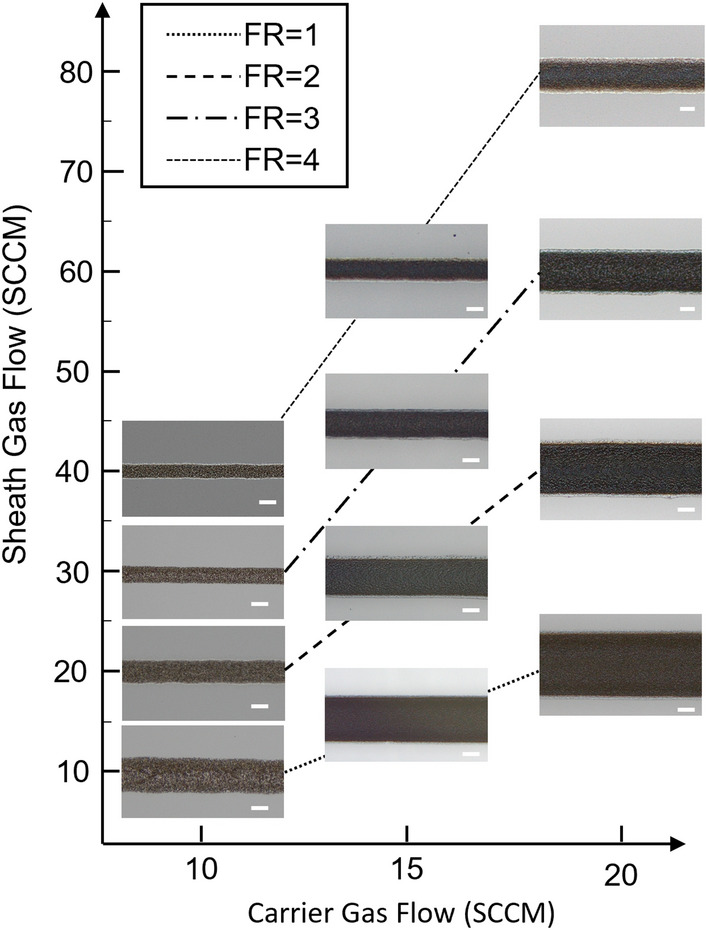
Optical microscopic micrographs of SPIONs lines printed on glass slides, illustrating the trend of the changes in line width by increasing the focus ratio (FR = sheath gas flow rate/carrier gas flow rate) for different carrier gas flow rates. Lines printed at the same focus ratio are grouped together. Scale bar 20 µm.

Within this framework, they evidenced that the thickness of the printed line increase with increasing FR, while the width decrease. Our previous work on the aerosol jet printing of PEDOT:PSS micro-features also confirmed these results^17^. In this work, the carrier and the sheath flow rates were varied to print lines with different widths to present the capability of the system (see “Materials and methods” section for details). Nozzle size, scanning speed and working distance were fixed respectively at 100 µm, 2 mm/sec and 2.5 mm. The quality of the lines was initially checked by optical microscopy and the optimal window settings for the deposition have been determined by observing at what point did increasing/decreasing carrier gas flow rate and focusing ratio defects begin to appear. In particular, below a carrier gas flow rate of 10 SCCM the deposited ink is insufficient to produce a continuous line, while above 20 SCCM the excess ink deposited causes lines with irregular bulges. As regard the focus ratio, although increasing FR results in narrower lines with more distinct edges, the focus ratio cannot be infinitely altered; previous work has shown that beyond a certain threshold a further increase in the FR no longer improves resolution but once again causes poorly defined lines^50,51^. For our system (spanning. the combination of ink, nozzle diameter, and atomization method), we found this threshold to be 4. In conclusion, acceptable deposition occurred for carrier flow rates between 10 and 20 SCCM with focus ratios between 1 and 4, resulting in printed lines with defined edges and reduced overspray (Fig. 3). It was confirmed that increasing the carrier flow rate results in wider lines, while increasing the FR yield narrower lines.

**Figure 4:**
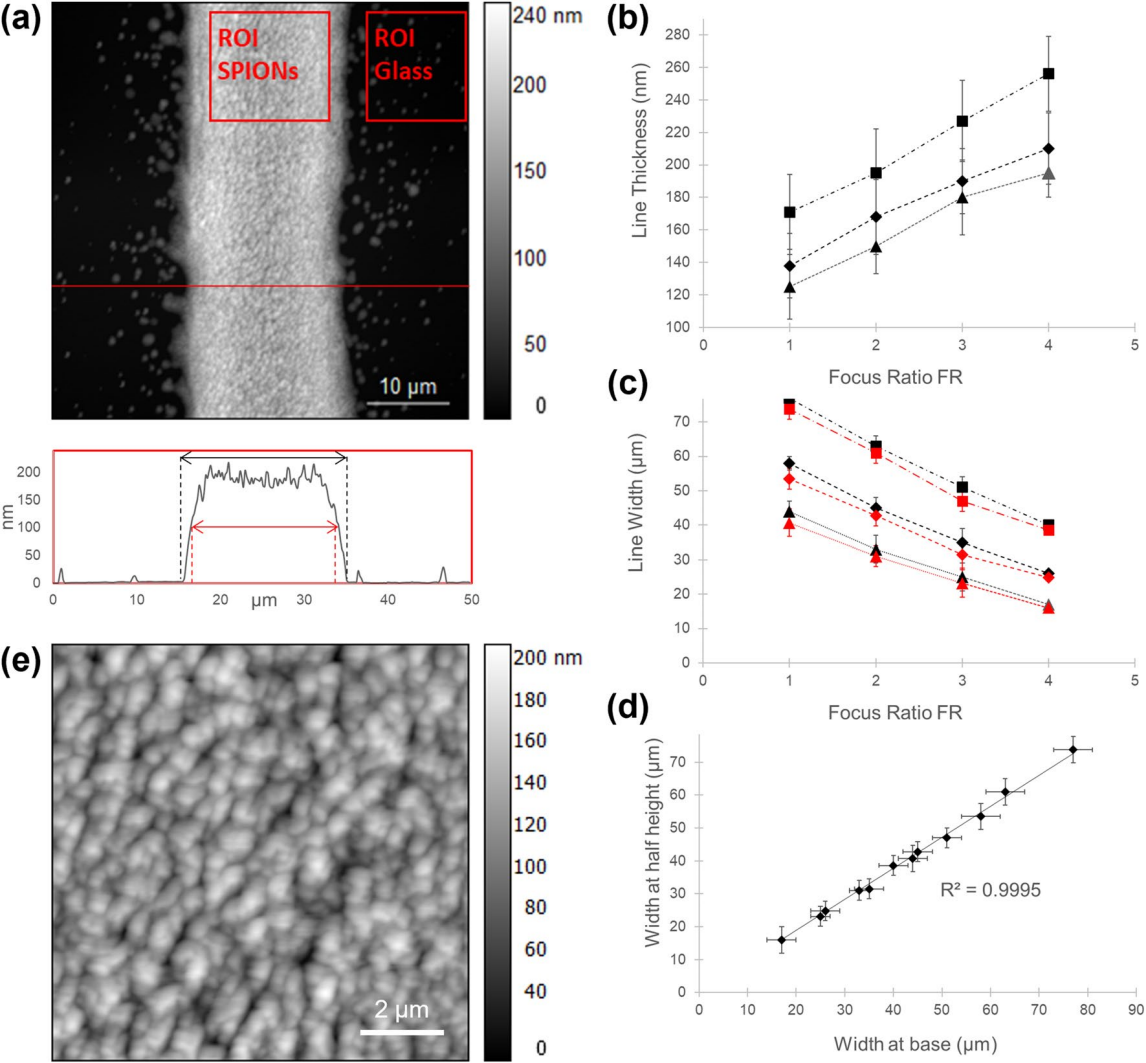
AFM analysis of SPIONs lines printed on glass slides. (**a**) Example of an AFM scan across a SPIONs printed line (carrier gas flow 10 SCCM, sheath gas flow 40 SCCM, FR = 4) and its cross-sectional profile along the horizontal red line. The line average thickness was evaluated as the difference between the average height of an ROI selected on the line surface (ROI SPIONs) and the average height of the ROI on the glass slide (ROI Glass). The width at base (black) and the width at half height (red) are also shown. (**b**) Line thickness plotted against focus ratio for different carrier gas flow rate: (Black Triangle) 10 SCCM, (Black diamond) 15 SCCM, (Black square) 20 SCCM. Error bars indicate the standard deviation of line height in AFM scans (RMS roughness). (**c**) Width at base (black) and the width at half height (red) plotted against focus ratio for different carrier gas flow rate: (Black Triangle)10 SCCM, (Black diamond)15 SCCM, (Black square) 20 SCCM. (**d**) Line width at half height versus line width at base. (**e**) 10 μm × 10 μm surface scan (carrier gas flow 10 SCCM, sheath gas flow 40 SCCM, FR = 4).

Atomic force microscopy (AFM) was then used to assess the average thickness, the width at base, the width at half height, and the roughness of the magnetic lines deposited via AJP onto glass substrates (see “Materials and methods” section for more details). A representative example of these measurements is reported in Fig. 4a, showing the AFM topography image across the edges of a printed line, and its cross-sectional profile along the horizontal line. The line profile presented negligible concave shape at the central area, confirming that the addition of the 10% v/v of terpineol as co-solvent had a major effect on negating the coffee-ring depositions at the edge, which is frequently observed for fabricated lines based on jet printing technologies^52^. As displayed in Fig. 4b, the average thickness of the lines ranged from 125 ± 23 to 256 ± 29 nm, confirming that for the same carrier flow the average thickness increases with respect to the focus ratio (FR), as expected from the AJP depositions of nanoparticles suspension^50^. It is noteworthy that the thickness of the printed lines is in the nanometre regime; the deposition of ultra-thin magnetic patterns allows the surface decoration of soft/flexible micro/nano-structures without affecting their deformability, as demonstrated below for PLLA nanofilms. In this particular application, a thick patterning might impair this flexibility or prevent the structural features being realised. However, in different applications where more magnetic material deposition is needed, the thicknesses of the SPIONs patterns could be varied by controlling the number of print passes, using a multilayer approach^53^. Multiple print passes could be used to finely control the thicknesses from hundreds of nanometres to several micrometres.

The influence of the FR on both width at the base and width at half height (the latter highlighted in red) is shown in Fig. 4c; as expected, the trend of decreasing line width with increasing FR was confirmed. A strong linear relationship (R^2^ = 0.9995) between line width at the base and half height (Fig. 4d) was found, suggesting that the printed lines have a uniform geometry and a consistent print profile across the different line sizes^17^. From width measurements, it was determined that the aerosol-jet set up is capable of printing magnetic patterns with well-defined edges with feature sizes down to 17 μm. This result demonstrated that the printing resolution achievable with AJP technology for magnetic materials is higher compared to other digitally driven techniques, such as ink-jet printing^12^. The surface of samples was then scanned over 10 μm × 10 μm areas to investigate surface topology and measure the surface roughness, estimated as the average absolute deviation from the mean height value. A representative example of these measurements is reported in Fig. 4e. AFM topography images confirmed that, after the printing and the evaporation of the solvents, SPIONs aggregate in grains with a dense and homogeneous distribution across the line. Depending on the application, the inhomogeneity of the line roughness can potentially affect the performance of printed devices. The limited coffee-ring effect, evidenced in Fig. 4a, assists a good homogeneity of nanoparticles distribution along the line profile. Consequently, in the case presented the difference in the line edge roughness with respect to the surface roughness is negligible. It was found that grain size and surface roughness did not vary significantly with FR, but slightly increase with the carrier gas flows, as reported in Table 1. The result is in agreement with previous studies on printed silver nanoparticle inks, which demonstrated that grain size and surface roughness of the deposited lines mainly depends on material formulation and solvent properties (boiling point, surface tension, polarity) and drying conditions (temperature and treatment duration)^54,55^.

**Table 1:** Roughness of SPIONs tracks.

Focus ratio	Roughness (nm)		
	Carrier gas flow 10 SCCM	Carrier gas flow 15 SCCM	Carrier gas flow 20 SCCM
1	23.6	27.8	29.5
2	25.2	27.3	28.9
3	24.3	25.4	29.2
4	23.8	28.2	28.3

The roughness was determined from $R_{a} = \left( 1 \right)/\left( N \right)\sum \left| {Z_{i} - \overline{z}} \right|$ from 10 μm × 10 μm AFM scans.

**Figure 5:**
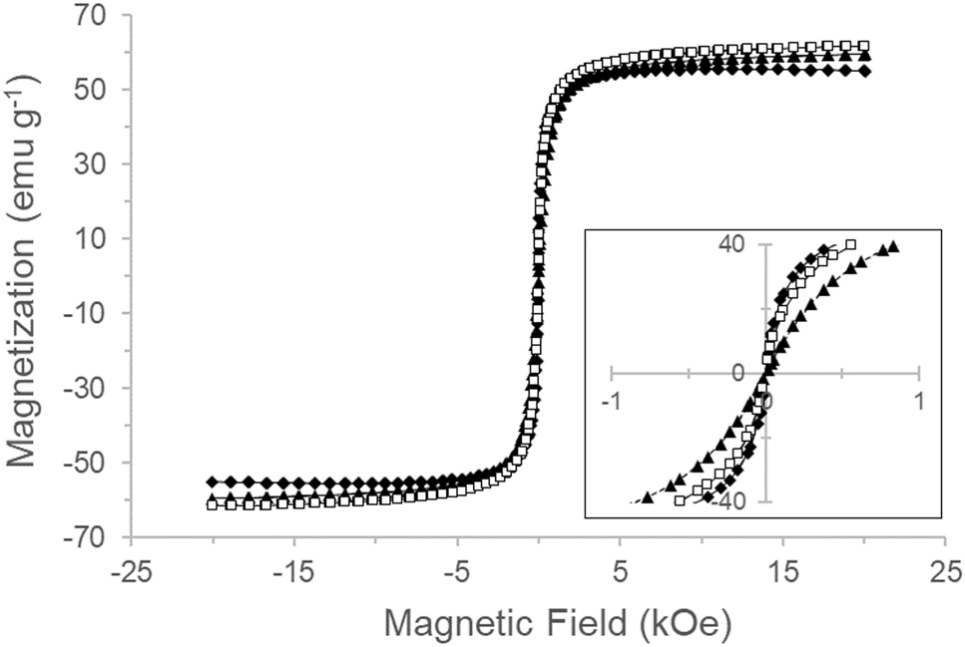
Magnetization hysteresis plots of pristine SPIONs (Black Triangle) and aerosol jet printed SPIONs patterns on silicon wafer (Black diamond) and PTFE tape (White square).

To characterize the magnetic response of the aerosol jet printed SPIONs patterns, their magnetization hysteresis was evaluated by superconducting quantum interference measurement device (SQUID) and compared to the magnetization hysteresis of pristine SPIONs. In fact, SPIONs aggregation in grains during the processing could in general modify their collective magnetic behaviour, depending on the strength of interparticle interactions^56^. Figure 5 displays the hysteresis loops for pristine SPIONs and aerosol jet printed patterns on different substrates measured at 300 K. All hysteresis loops showed no remanence or coercivity, suggesting all the particles are in the superparamagnetic regime, as expected for weakly interacting nanoparticle assemblies^57^. The observed behaviour of the printed SPIONs strictly resembles that measured for the pristine SPIONs, indicating that the magnetic properties were successfully transferred to the printed patterns without significant modification due to the processing.

**Figure 6:**
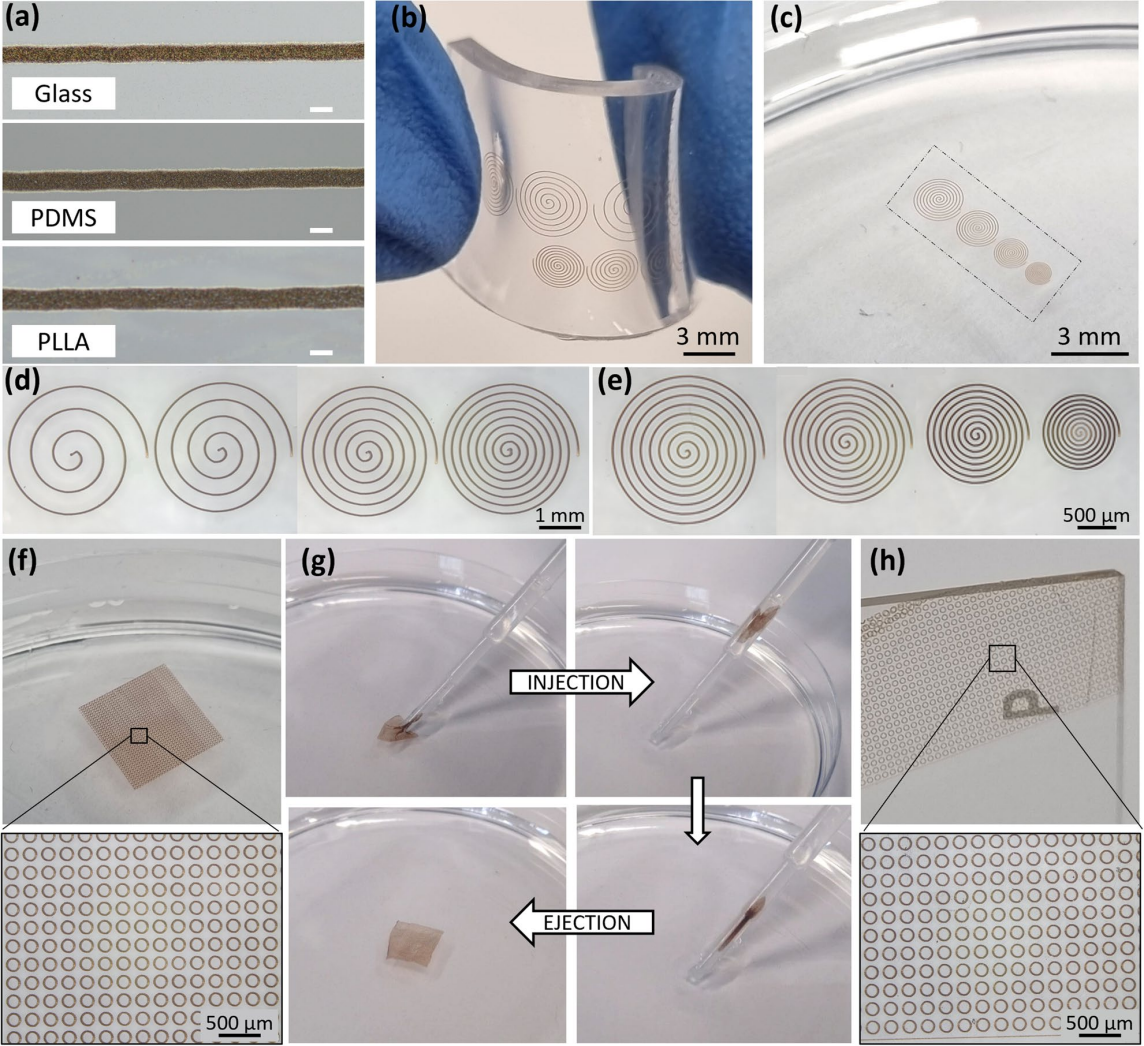
(**a**) Comparison of optical microscopic micrographs of SPIONs lines printed on top of a glass slide, a PDMS film and a PLLA nanofilm (carrier gas flow 10 SCCM, sheath gas flow 40 SCCM, FR = 4). Scale bar 20 µm. (**b**) A PDMS film with patterned surface. (**c**) A patterned PLLA nanofilm floating over the water surface after the release from the fabrication substrate (the edges of the nanofilms are highlighted by a dashed line). Optical microscopic images of spirals with different pitch and external radius printed on PDMS (**d**) and PLLA (**e**). (**f**) Released patterned nanofilm (15 mm × 15 mm) floating over the water surface and optical microscope magnification. (**g**) Patterned nanofilm injection (top) and ejection (bottom) sequence. (**h**) Patterned nanofilm collected and dried on a glass slide and optical microscope magnification after 10 injection and ejection cycles.

To demonstrate some of the potential of this AJP technique, the ability to print magnetic patterns on materials widely used in biomedical engineering and soft robotics was investigated. Figure 6a reports optical micrographs of magnetic lines printed on a glass slide, a PDMS film and a PLLA nanofilm using the same process parameters. Good quality printed lines with well-defined edges can be observed for all the substrates. The specifics of the line geometry, such as the average height, width at base, width at half height, and surface roughness are subject not only to influences from the material formulation and process parameters, but also material/surface interaction and drying characteristics. In particular, the surface properties of the substrates, including roughness and surface energy, influence the adhesiveness and wetting characteristics of the magnetic ink. The different surface properties and consequent ink spreading result in lines with slightly different widths for the different substrates printed using the same processing parameters, as evidenced by optical microscope images reported in Fig. 6a. In particular, printing on PDMS and PLLA produces lines that are wider than on glass, ranging from 17 to 25 μm. For PDMS this can be attributed to the plasma treatment for 60 s applied to the substrates immediately prior the AJP deposition which is required to promote adhesion and assist the printing of a uniform line on these substrates. This is supported by the previous report that plasma treatment of the substrate’s surface prior to AJP increases material spreading and adhesion^14^. This slight variation between the substrates is reflective of these different processing requirements, but is one that is recognised and can be mitigated against if required by a certain application. In this paper we have, for the first-time, demonstrated that the AJP technique can be successfully used to freely deposit micro-scale magnetic patterns in the region of 20 μm on different material substrates.

In order to illustrate the high flexibility and scalability afforded by this technology, bespoke freeform patterns were then generated using the SPIONs formulation on the surface of PDMS films and PLLA nanofilms (Fig. 6b,c) and the corresponding optical microscopic images are shown respectively in Fig. 6d,e. The patterns were selected to demonstrate the geometrical shaping capabilities, the printing resolution, and flexibility of the process. Consequently, this is demonstrated through a spiral geometry with different pitches and external radii, the track/gap relationship provided by the resolution, and explanation of the rapid alteration and tunability of these designs and the dimensions of the patterns. The individual spirals shown in Fig. 6d were printed within a period of 13 s (left) to 28 s (right). The print time for the entire set of patterns was below 80 s. The high flexibility, scalability, and high production speed afforded by the AJP technology means a vast range of other patterns could be rapidly created which is key contribution of the research.

SPIONs patterned PLLA nanofilms were then selected for further investigation. In general, thanks to the combination of nanometre thickness and macroscopic size, polymeric nanofilms possess unique physical properties, such as high flexibility, injectability, and noncovalent adhesiveness, which are beneficial for many applications^45^. Within this framework, it is important to verify that the SPIONs surface patterning of PLLA nanofilms does not affect these peculiar characteristics. Figure 6f shows a picture of a 15 mm × 15 mm PLLA nanofilm patterned over the entire surface with SPIONs circles of 200 µm in diameter and a pitch of 300 µm. Due to the hydrophobicity of PLLA, after the dissolution of the PVA sacrificial layer and the release from the fabrication substrate, the nanofilm floated over the water surface (Fig. 6f). After the addition of more PVA (0.1 wt %) to the water, in which PVA was acting as a surfactant, the manipulation of the patterned free-standing nanofilm with a pipette was possible, injecting and ejecting the nanofilm multiple times without breaking it (Fig. 6g). Even after manipulation, the patterned nanofilms spread completely unfolded in the suspending medium, confirming that the SPIONs patterning did not affect its flexibility and injectability. After 10 injection and ejection cycles, the patterned nanofilm was collected and dried on a glass slide, to which it adhered by physical adhesion, and its surface was observed with the optical microscope (Fig. 6h). No distortions of the SPIONs patterns due to injection and ejection cycles were observed, confirming the good adhesion of SPIONs to the nanofilm surface and the preserved flexibility of the structure.

**Figure 7:**
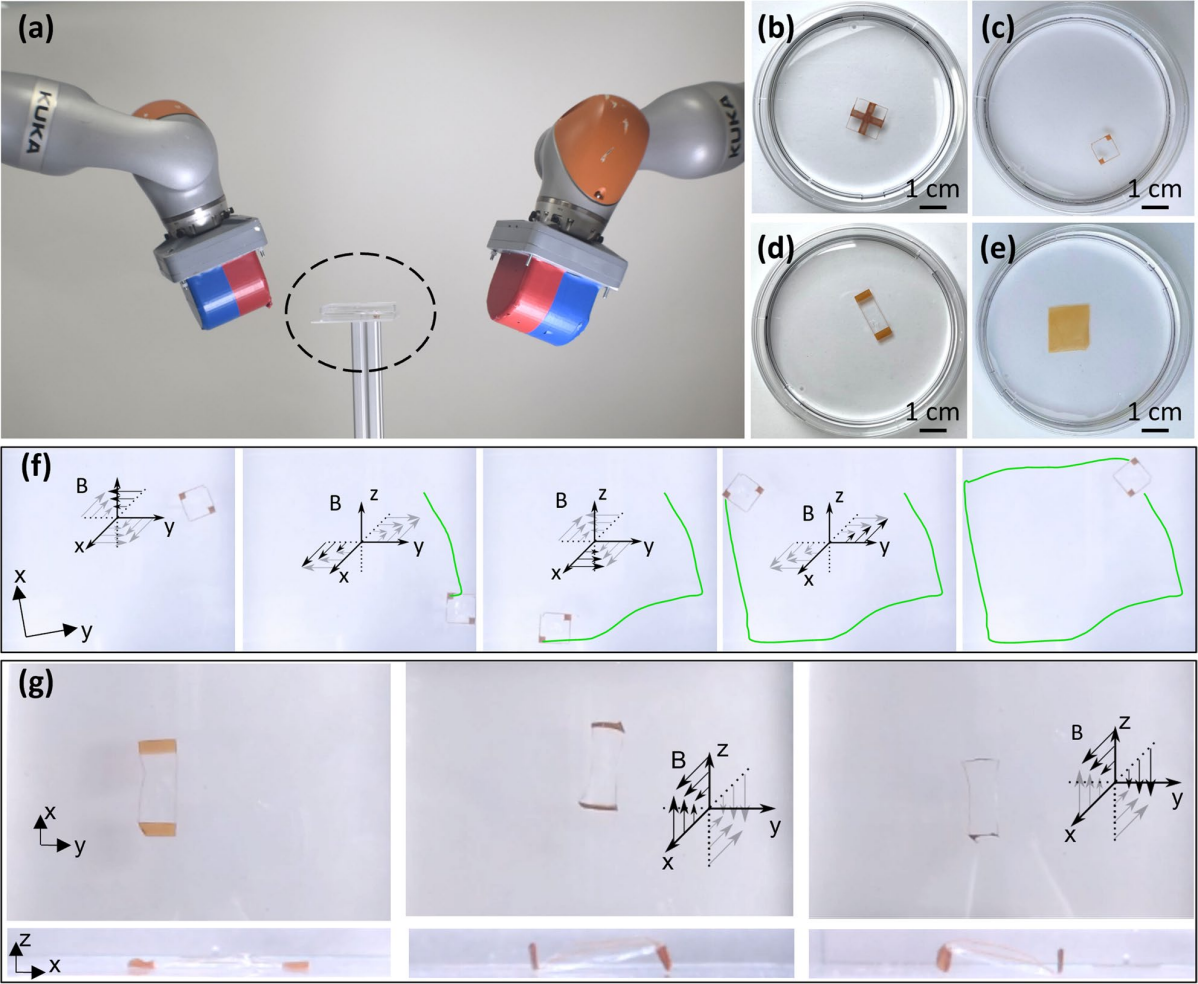
(**a**) The dual External Permanent Magnet (dEPM) platform used for the magnetic manipulation experiments. The petri dish containing the magnetic patterned nanofilm floating on the surface of the water is highlighted by a dashed line. Different patterns tested: (**b**) cross-patterned nanofilm; (**c**) two-corners patterned nanofilm; (**d**) two-side patterned nanofilm. (**e**) A homogeneous nanofilm used as a control. (**f**) A two-corners patterned nanofilm performing a planned sequence of rotations and translations along a square path. (**g**) A two-side patterned nanofilm showing 2D-to-3D shape morphing by out-of-plane bending: starting from the flat position (left) the nanofilm changes shape into a mirrored N-shape (central) and an N-shape (right) according to the applied magnetic field gradients.

Finally, the proposed patterning method was applied to the development of patterned magnetic nanofilms with enhanced locomotion capabilities. In the past few years, magnetic responsive nanofilms prepared by spinning a PLLA/CHCl_3_ solution containing SPIONs and with a homogeneous distribution of magnetic nanoparticles throughout their body were proposed, and their remote-controlled manipulation with external magnetic fields was demonstrated by dragging the nanofilms onto the water surface with the use of a permanent magnet^45^. In this present work, we investigated the magnetic manipulation of patterned nanofilms using magnetic field gradient generated by the dual External Permanent Magnet (dEPM) robotic platform shown in Fig. 7a (see “Materials and methods” section for details). Different patterned nanofilms with milli-scale designs were tested (Fig. 7b–d); note that if the maximum SPIONs line size obtained with AJP is ≈ 80 μm, wider patterns can be obtained printing multiple connected parallel lines with a small shift of around 80 μm in between each line. Thus, the width of the patterns can be controlled by the number of parallel lines, to gradually build the width up to the millimetre scale. A homogeneous nanofilm was used as a control (Fig. 7e). Compared to lower resolution patterning methods, such as screen printing, the main advantage of the presented patterning method is that AJP is a maskless deposition method which allows higher flexibility, iterative design changes and the freeform fabrication of the magnetic microfeatures. Moreover, another advantage compared to screen printing, is also the efficiency of material usage, as the material is only printed where required, with reduction in material waste which is particularly significant in the materials concerned. The cross-pattern nanofilm (Fig. 7b) was selected for the first set of experiments in order to confer to the nanofilm the ability to rotate under the action of the magnetic field gradient. In contrast to the static behaviour of the homogeneous nanofilm, the cross-pattern design demonstrated 160° anticlockwise rotation (within 8 s) followed by a 180° clockwise rotation (within 5 s) when subjected to planar gradient fields of 300 mT/m (Video S1 in Supporting Information). These bi-directional rotations were achieved by inverting the direction of the magnetic field while maintaining constant field gradients; generating opposite forces. The difference in rotation rate can be attributed to several factors, such as the nanofilm’s initial state, small inaccuracies in the nanofilm’s position within the workspace, and lastly, the magnetic field gradients generated by the EPM when moving into their initial position. Rotation was subsequently combined with controlled translation through manipulation of a fabricated sample with two magnetic corners (Fig. 7c). The sample was controlled to translate and rotate along a squared path (see Fig. 7f, see also Video S2, Supporting Information) via a sequence of symmetric planar field gradients. Given the geometric pattern of the nanofilm, the two magnetic corners are exposed to different magnetic field intensities, leading to a differential in magnetic moment intensity. This translates into differing magnetic forces which enables the sample to rotate as it translates. By alternating the direction of the gradient (300 mT/m) and the field (up to 15 mT), as illustrated in Fig. 7f, the nanofilm was able to travel a total length of 25 cm in 240 s along a squared path. Finally, 3D shape morphing was shown by inducing out-of-plane magnetic forces on a two-sided patterned nanofilm (Fig. 7d). By keeping an out of plane magnetic field gradient constant at 300 mT/m and inverting the perpendicular magnetic field direction of up to an absolute value of 6 mT, the nanofilm conformed to an N-shape and a mirrored N-shape in 9 s. Additionally, planar forces were also induced leading to translation of the sample (see Fig. 7g, see also Video S3, Supporting Information). For the presented test cases, the nanofilm’s pattern plays an essential role in the manipulation and shape morphing capabilities. Patterns will experience differential magnetic moments across their geometry when under a magnetic field gradient, leading to different manipulation and morphing behaviours. Ultimately, the specific application for such nanofilms will dictate the pattern required for enhanced manipulation and manoeuvrability.

## Conclusions

In this work, we have explored for the first time the potential of AJP as a digitally driven, non-contact and mask-less printing technology to realize magnetic patterns at micron-scales onto different substrates. We formulated a suitable magnetic ink (90% v/v toluene, 10% v/v terpineol, 20 mg/ml EMG1300M SPIONs) capable of producing magnetic micro-features with a minimum width < 20 μm. The entire manufacturing process is digitally driven, thereby providing the capability to rapidly alternate and produce different designs, and to do so within time and cost boundaries that would be unachievable by template-based manufacturing approaches. Micro-patterns of SPIONs were successfully printed onto both rigid as well as soft and flexible materials commonly used in soft robotics and biomedical engineering applications, such as PDMS films and PLLA nanofilms.

We believe that the use of this scalable, accurate and versatile digitally driven processing technology could pave the way towards new technological and biomedical applications that require high yield and rapid patterning of magnetic material with micro-scale resolution and over large areas. As a first proof of concept, we presented the possibility of using AJP to create micro/milli-scale magnetic patterns on top of PLLA nanofilms without affecting their particular characteristics, such as high flexibility and injectability. Moreover, thanks to the asymmetrical patterning of SPIONs, patterned nanofilms exhibited enhanced magnetic controllability when compared to homogenous nanofilms, showing that they can not only be dragged across the workspace but also allow rotation and 3D shape morphing. Patterned nanofilms also open other application routes in the biomedical field related to homogeneous nanofilms. Further studies will be required depending on the specific biomedical application.

## Supplementary Information

Supplementary Video 1.

Supplementary Video 2.

Supplementary Video 3.

## Data Availability

The data that supports the findings of this study are available from the corresponding author on request.
